# Optimising the consent process in severe traumatic brain injury trials

**DOI:** 10.1186/cc9735

**Published:** 2011-03-11

**Authors:** LJ Murray, D Cooper, JV Rosenfeld, Y Arabi, A Davies, P D'urso, T Kossmann, J Ponsford, P Reilly, I Seppelt, R Wolfe

**Affiliations:** 1 The Alfred Hospital, Melbourne, Australia; 2 King Fahad National Guard Hospital, Riyadh, Saudi Arabia; 3 Epworth Healthcare, Melbourne, Australia; 4 Monash University, Melbourne, Australia; 5 Adelaide University, Adelaide, Australia; 6 Nepean Hospital, Sydney, Australia

## Introduction

Severe traumatic brain injury (TBI) is a condition often associated with grave consequences and it remains a major public health problem globally. Clinical trials to improve management and treatment of this condition are a necessity; however, there are many issues that impact on the design and conduct of such trials including the complex and sensitive issue of consent. Obtaining consent for severe TBI trials is inherently complicated and difficult because the family are being asked to make an informed decision when they are shocked, anxious, grieving and frequently physically exhausted. We established a process during the DECRA trial to minimise the difficulties and to ensure that consent was obtained with sensitivity and in an informed manner.

## Methods

The DECRA trial is a prospective randomised trial of 155 patients from Australia, New Zealand, and Saudi Arabia. Patients with severe traumatic brain injury and refractory intracranial hypertension were randomly assigned to receive either a decompressive craniectomy or to continue with standard medical management. Surrogate consent was obtained prior to randomisation and all participating hospitals had obtained approval from their Human Research & Ethics Committee.

## Results

Guidelines for obtaining consent were included in the protocol and manual of operations, and were discussed at the investigators' meetings. The guidelines highlighted the importance of early communication with the patient's medical team regarding possible recruitment into the trial, updating the family about the patient's condition prior to the consent discussion, following a basic script to ensure all aspects of the trial were covered in the discussion, allowing time for the discussion including follow-up discussions and listening carefully to the family's questions. DECRA commenced recruitment in 2003 and the last patient was enrolled in April 2010; 168 consent discussions were held with a 92% consent rate.

## Conclusions

Consent rates in brain injury studies in the critical care setting can be optimised by following a protocolised consent process.

